# *Potentillae argenteae herba*—Antioxidant and DNA-Protective Activities, and Microscopic Characters

**DOI:** 10.3390/antiox14040487

**Published:** 2025-04-18

**Authors:** Tsvetelina Andonova, Yordan Muhovski, Samir Naimov, Elena Apostolova, Silviya Mladenova, Ivayla Dincheva, Vasil Georgiev, Atanas Pavlov, Rumen Mladenov, Ivanka Dimitrova-Dyulgerova

**Affiliations:** 1 Department of Botany and Biological Education, Faculty of Biology, University of Plovdiv “Paisii Hilendarski”, 4000 Plovdiv, Bulgaria; ts_andonova@uni-plovdiv.bg (T.A.); rummlad@uni-plovdiv.bg (R.M.); ivadim@uni-plovdiv.bg (I.D.-D.); 2 Biological Engineering Unit, Life Sciences Department, Walloon Agricultural Research Centre, 5030 Gembloux, Belgium; 3 Department of Molecular Biology, Faculty of Biology, University of Plovdiv “Paisii Hilendarski”, 4000 Plovdiv, Bulgaria; naimov0@uni-plovdiv.bg (S.N.); eapostolova@uni-plovdiv.bg (E.A.); 4 Department of Human Anatomy and Physiology, Faculty of Biology, University of Plovdiv “Paisii Hilendarski”, 4000 Plovdiv, Bulgaria; silviamladenova.sm@uni-plovdiv.bg; 5 Department of Agrobiotechnologies, AgroBioInstitute, Agricultural Academy, 1164 Sofia, Bulgaria; ivadincheva@abi.bg; 6 Laboratory of Cell Biosystems, Institute of Microbiology, Bulgarian Academy of Sciences, 139 Ruski Blvd., 4000 Plovdiv, Bulgaria; vasgeorgiev@microbio.bas.bg (V.G.); a_pavlov@uft-plovdiv.bg (A.P.); 7 Department of Analytical Chemistry and Physical Chemistry, Technological Faculty, University of Food Technologies, 4002 Plovdiv, Bulgaria; 8 Department of Medical Biochemistry, Faculty of Pharmacy, Medical University of Plovdiv, 15A Vasil Aprilov Blvd., 4002 Plovdiv, Bulgaria

**Keywords:** *Potentilla argentea*, aerial parts, ethanol dry tincture, GC/MS identification, phenolic compounds (HPLC), in vitro antioxidant and DNA protective capacity, microscopic characters of herbal drugs

## Abstract

Antioxidants from natural sources are essential for the development of new therapeutics to improve human health. The objects of study are the aerial flowering parts of *Potentilla argentea*, a plant species known in traditional medicine for the astringent, hemostatic, wound-healing, and anti-inflammatory effects of its rhizomes. A *Potentillae argenteae herba* ethanol dry tincture was chromatographically analyzed (GC/MS, HPLC) and its antioxidant (ABTS, DPPH, CUPRAC, FRAP assays) and DNA nicking protection potentials were evaluated. The eighteen volatiles were identified by GC/MS, where the predominant components were n-nonacosane (39.38 mg/g dt), squalene (28.88 mg/g dt), n-tricosane (18.36 mg/g dt), ethyl oleate (15.24 mg/g dt), and n-pentacosane (10.60 mg/g dt). A high content of total polyphenols was obtained (440.78 mg GAE/g dt), and HPLC analysis identified two flavonoids and three phenolic acids, of which rosmarinic acid and rutin were above 10 mg/g dt. The tincture exhibited strong antioxidant activity by all four methods, especially CUPRAC assay (8617.54 μM TE/g). DNA protective activity against oxidative damage and microscopic identification of *P. argenteae herba* powder were established for the first time. Therefore, the tincture could be incorporated into phytopreparations for the treatment of human diseases caused by reactive oxygen species.

## 1. Introduction

Medicinal plants have been used to treat numerous human diseases for centuries [1]. Herbal extracts obtained from their leaves, stems, shoots, roots, rhizomes, and other plant parts are the basis of traditional medicine [2]. The identification and isolation of the biologically active components of extracts are important and essential steps in the discovery and construction of new, more effective pharmaceuticals. Plant species of the genus Potentilla (Rosaceae family), widely used in folk medicine in the treatment of lung and stomach inflammation, bleeding, some forms of cancer and diabetes, mouth ulcers, toothache, sore throat, dysentery, jaundice, wound healing, etc., are distinguished by their powerful healing potential [1,3]. Their plant extracts are defined as being reliable for the preparation of a variety of pharmaceutical drugs for the treatment of diseases ranging from simple infections to oncological diseases [1]. Individual bioactive ingredients in them show antioxidant, antitumor, antimicrobial, anti-inflammatory, enzyme-inhibitory, antidiarrheal, and other effects [4,5,6,7,8,9]. Researchers define some representatives of the genus Potentilla as a kind of “chemical storehouse” for biologically active compounds such as phenols (pyrogallol, phloroglucine, and pyrocatechin), saponins, procyanidins, triglycerides, macro- and microelements, and essential oil [7].

*Potentilla argentea* L. (common name silver cinquefoil) is a perennial herb, native to Europe, including Bulgaria’s rocky areas and pastures, up to 1700 m above sea level. The plant species is known in Bulgarian folk medicine for the astringent, hemostatic and anti-inflammatory effects of its rhizomes, due to the tannin content [10]. The species is part of the medicinal flora of different cultures, where its plant extracts are used as a healing agent for skin irritations, dermatitis, allergic rashes, treatment of digestive disorders, etc. [6,11,12].

The manifested biological activities are the reason for the emergence of interest in the study of the chemical composition of *P. argentea*. Total polyphenols represent part of the bioactive compounds and have been reported for their various extracts (aqueous, methanol, and ethyl acetate) from above-ground [5,6,13,14] and underground [9] parts. There is evidence of the presence of individual phenolic components such as quercetin, kaempferol, isoquercitrin, rutin, tiliroside, and methyl brevifolincarboxylate in the species’ chemical composition [3,8]. In separate studies on the phytochemical profile of a methanol extract from above-ground parts of the plant species, ellagitannins, flavonol glycosides, triterpene constituents, etc., were identified [13]. An ethyl acetate extract of *P. argenteae herba* contains quercetin 3-glucuronide [15], and aqueous-epigallocatechin gallate, galloyl-glucose, tiliroside, and quercetin derivatives [14]. MS and UV-Vis data of acetone extracts reveal the presence of gallic acid and its derivatives, catechin, tiliroside, brevifolin carboxylic acid, isoramnertine, and other phenolic compounds [6]. The roots and leaves of *P. argentea* are a source of macro- and micronutrients (Ca, K, Si, Fe, Mg, Mn, Al, S, P, Cl, Ba, Zn, Mn, Na, etc.), and the composition of a methanol extract from the roots contains squalene, cis-13-octadecenoic acid, methyl β-D-glucopyranoside, and other compounds [7]. According to other authors, the flavonoids rutin and catechin are among the main phenols of the underground parts [9].

For extracts of *P. argentea*, antioxidant, enzyme-inhibitory, cytotoxic, and antiproliferative activities have been indicated [4,8,13,16,17]. Individually identified compounds (catechin and rutin) in methanol extracts from the roots have been associated with the observed antioxidant activity [9]. Flavonoid glycosides and catechin dimers have been found in aqueous extracts that have shown anti-urease activity [14]. Anticarcinogenic activity against colorectal carcinoma has been reported for its acetone extracts [6]. Some authors propose extracts of *P. argentea* roots for the needs of medicine and pharmacy as a reliable antimicrobial agent against clinical isolates of *Staphylococcus aureus* and *Escherichia coli* [7]. Glycosidic flavonoid tiliroside and methyl brevifolincarboxylate isolated from *P. argentea* have been studied for DNA topoisomerase inhibitory activity [4].

The information reported so far shows that the species has valuable bioactive components that determine various pharmacological effects. Mainly, its methanol, acetone, ethyl acetate, and aqueous extracts have been studied. In the initially obtained extracts, the phytocomponents are in a diluted form, and in order to increase their healing power, as well as for better storage, easier transport, and other benefits, it is necessary to concentrate them. Many researchers have focused on the development of methods to concentrate extracts (solvent evaporation at low temperature, vacuum drying, pressure filtration, etc.), striving to keep the medicinal properties unchanged [2]. Due to the lack of data on the ethanol extract from the *P. argenteae herba*, and the fact that ethanol is one of the most used solvents for the extraction of bioactive components from herbal drugs, we decided to investigate this type of extract. For all the reasons mentioned above, we chose to analyze the composition and activity of a concentrated ethanol extract, or the so-called dry tincture of dried aerial parts. Furthermore, phytochemical and biological studies of *P. argentea* have not been conducted in Bulgaria until now. All this determined the following purpose of the study, specifically chromatographic (HPLC, GC/MS) identification of the chemical composition of ethanol dry tincture of *P. argentea herba*, study of its biological activities (antioxidant and DNA nicking protection), and determination of the main diagnostic microscopic characters of the plant drug’s powder.

## 2. Materials and Methods

### 2.1. Chemicals and Reagents

The reagents for the chromatographic (HPLC and GC/MS) and spectrophotometric analyses to determine total polyphenols and antioxidant activities were supplied by Sigma-Aldrich (Steinheim am Albuch, Germany). Information about their catalog numbers can be found in our previous publication [18]. Pyridine and N,O-bis(trimethylsilyl)trifluoroacetamide (BSTFA) and n-nonadecanoic acid analytical standard 646-30-0 (Supelco) were purchased from Sigma-Aldrich (Steinheim am Albuch, Germany). Acetonitrile, methanol, acetic acid, and ethanol were utilized as HPLC-grade solvents; rutin, quercetin, kaempferol, hesperidin, (+)-catechin, (−)-epicatechin; and gallic, protocatechuic, vanillic, syringic, *p*-coumaric, salicylic, chlorogenic, ferulic, rosmarinic, and caffeic acids served as analytical standards. For the DNA in vitro tests, the reagents were purchased from the previously mentioned company, in addition to agarose SPI and TBE buffer from Duchefa Biochemie (Haarlem, The Netherlands). For the microscopic analysis of the plant samples, chloral hydrate ≥ 98.5% was used as a clearing agent, (Ph. Eur. CAS No. 302-17-0), obtained from Carl Roth GmbH (Karlsruhe, Germany).

### 2.2. Plant Material Collection, Identification and Obtaining the Dry Tincture

**Figure 1:**
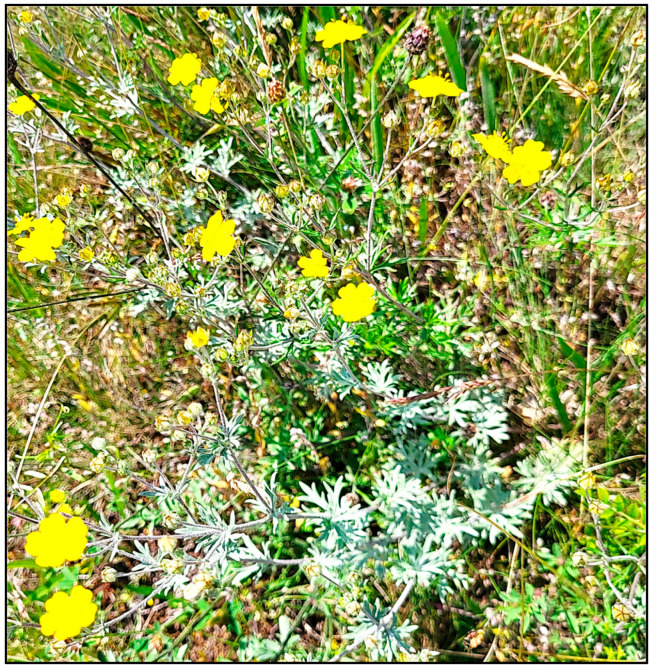
*Potentilla argentea* in its natural habitat (author’s photograph).

Above-ground plant parts of *P. argentea* were collected during the period of active flowering (July 2023) from the territory of the Devin municipality, Rhodope Mountains, Bulgaria (N 41°46′55″ E 24°22′13″; 1362 m above sea level). A photograph of the species in its natural habitat is shown in Figure 1. The systematical identification of the plant materials was performed at the Department of Botany, University of Plovdiv “Paisii Hilendarski”, Bulgaria (by one of the authors—I.D-D.). A voucher specimen (No 063404) was deposited in the herbarium at the Agricultural University (SOA), Bulgaria.

**Figure 2:**
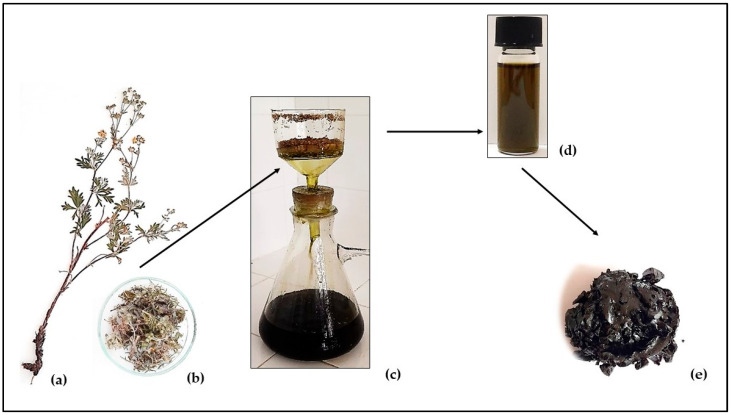
A scheme of the main steps in the process of obtaining the tincture: air-dried whole (**a**) and cut (**b**) above-ground parts of *Potentilla argentea*; pressure extract filtration (**c**); tincture before vacuum drying (**d**); tincture after vacuum drying (dry tincture, DT) (**e**).

After collection, the plant material (100 g) was dried in the shade and at room temperature for about 20 days (Figure 2a,b), and then ground to a fine powder using an electric laboratory mill (GRINDOMIX GM 200, Retsch GmbH, Haan, Germany). The methodology for obtaining the tincture is a standard procedure, specified in the Pharmacopoeia, consisting in soaking one part of the plant in 10 parts pure 96% ethanol [19]. The duration of the extraction was 10 days (at room temperature; in the dark) with periodic stirring, followed by vacuum filtration (8–12 μm pore size filter, Figure 2c,d). The resulting tincture was additionally dried at 50 °C and 97 mbar (BUCHI R-300, Rotavapor, BÜCHI Labortechnik AG, Flawil, Switzerland, Figure 2e). Some of the main steps in the process of obtaining the tincture of *P. argenteae herba* are shown in Figure 2.

### 2.3. Gas Chromatography–Mass Spectrometry (GC-MS) Quantification of Chemical Composition

Quantities of 10 mg of dried extract, 100 μL pyridine, 100 μL BSTFA, and 50 μL of internal standard solution (nonadecanoic acid at 0.05 mg/mL in chloroform) were introduced into a glass vial. After vortex mixing of the sample, the vial was heated for 45 min at 70 °C in a heating block. The obtained samples were analyzed by gas chromatography–mass spectrometry (GC-MS). The chromatographic analysis was conducted using an Agilent 7890A gas chromatograph connected to a 5975C mass selective detector from Agilent Technologies. The separation was carried out on a J&W DB-5ms silica-fused capillary column (30 m × 0.25 mm) with a 0.25 µm poly(dimethylsiloxane) film (Agilent Technologies, Santa Barbara, CA, USA) as the stationary phase. Helium served as the carrier gas at a 1.0 mL/min flow rate. The injector and transfer line temperatures were both set to 250 °C. The oven temperature program started at 100 °C for 2 min, increased by 15 °C/min to 180 °C, then by 10 °C/min to 300 °C, where it was held for 10 min. A 1 µL sample was injected in split mode with a 10:1 ratio. The mass spectrometer operated in electron impact (EI) mode at 70 eV, scanning a range of 50 to 550 *m*/*z*.

To calculate the relative retention index (RRI) for each component, a series of aliphatic hydrocarbons (C8–C40) was analyzed under identical conditions. The identification of compounds was based on comparisons of retention indices and mass spectra with the NIST’08 database [20]. All compounds were verified through molecular ion peaks and analysis of their characteristic fragmentation patterns. The semi-quantification process was performed using internal normalization, where the area of each compound was measured. The sum of all compound areas equaled 100%.

### 2.4. Total Phenolic Assay and HPLC Quantification

The Folin–Ciocalteu method was used to analyze the total phenolic content of the investigated extract as described previously [21]. The analyses were performed in a 96-well plate by mixing 20 µL of extract (0.1 mg/mL in ethanol) with 180 µL of Folin–Ciocalteu reagent and adding 100 µL of sodium carbonate (7.5%) 2 min later. The reaction mixture was incubated (8 min at 37 °C), and the absorbance against a blank was read (λ = 750 nm) by a microplate reader (Multiskan FC, Thermo Fisher Scientific, Waltham, MA, USA). The results are expressed as gallic acid equivalents.

Quantification of individual phenolic compounds in extract was performed on an HPLC (Waters 1525) system with a UV-Vis detector (Waters 2487) as described previously [21]. In brief, the extract was dissolved in ethanol at a concentration of 2.5 mg/mL and filtered by a 0.45 μm syringe filter (Sartorius AG, Göttingen, Germany). The extract was injected (20 µL) into a C18 column (Supelco Discovery HS; 5 μm, 25 cm × 4.6 mm) (Merck KGaA, Darmstadt, Germany) and a gradient elution (1.0 mL/min flow rate) by mobile phase formed by mixing of 1% acetic acid (Solvent A) and methanol (Solvent B). The absorption at λ = 280 nm was used to detect gallic, protocatechuic, vanillic, syringic, *p*-coumaric, and salicylic acids, and (+)-catechin, (+)-epicatechin, and hesperidin. Rosmarinic, chlorogenic, caffeic, and ferulic acids, and rutin, quercetin, and kaempferol were detected at λ = 360 nm. Quantification was performed by retention times and calibration curves of external standards.

### 2.5. Determination of Biological Activities

#### 2.5.1. Antioxidant Activity Assays

The antioxidant potential of the dry tincture (DT) was evaluated by four different in vitro antioxidant tests, conducted according to Krasteva et al. [21], namely ABTS (2,2′-azino-bis (3-ethylbenzothiazoline-6-sulfonic acid)) and DPPH (1,1-diphenyl-2-picrylhydrazyl) radical scavenging assays, FRAP (ferric reducing antioxidant power) and CUPRAC (cupric reducing antioxidant capacity) assays. The reactions were performed in a 96-well plate by mixing 20 µL of the extract (0.1 mg/mL in ethanol) with 280 µL of the radicals (ABTS and DPPH) and FRAP reagent. For the CUPRAC assay, the extract was mixed with 70 µL copper (II) chloride (10 mM), 70 µL neocuproine (7.5 mM), 70 µL 1.0 M ammonium acetate buffer (pH 7.0), and 70 µL pure water. Changes in absorbance of the sample and blank (without extract) at a specific wavelength (λ = 734 nm—ABTS; λ = 515 nm—DPPH; λ = 593—FRAP; λ = 450 nm—CUPRAC) were read after a 15 min period of incubation (for ABTS and DPPH) and 10 min (for FRAP and CUPRAC) at 37 °C, and used to calculate the antioxidant activity of the sample. For the ABTS and DPPH assays, IC_50_ values were determined by plotting % radical inhibition vs. concentration (analyzed in concentrations of 50 to 300 µg/mL for extracts and from 10 to 100 µg/mL for standards).

All results are presented as micromoles Trolox (6-Hydroxy-2,5,7,8-tetramethylchromane-2-carboxylic acid) equivalents per gram extract by using a standard curve (50 to 200 µg/mL Trolox).

The butylhydroxytoluene (BHT) and L-ascorbic acid standards were used as positive controls.

#### 2.5.2. DNA Protective Capacity Assay

The DNA protective effect of the DT was analyzed using supercoiled pUC19 plasmid, purified from *E. coli* according to previously established protocols. [22,23]. The DT was initially dissolved in absolute ethanol, and further diluted in Milli-Q water to the test concentrations of (1 ng/mL; 0.1 ng/mL; and 0.01 ng/mL). Ten microliters from each solution was tested in Fenton’s reagent, supplemented with 200 ng supercoiled DNA. The reactions, set in a volume of 20 μL, were conducted at 37 °C for 30 min. Different concentrations of Trolox (25, 75, and 100 μg/mL) and water were used as positive and negative controls. The reaction products were analyzed on 1.0% agarose gel in 0.5 × TBE buffer under standard conditions. The amount of relaxed DNA was assessed by densitometry using the Gel Doc^TM^ EZ software -Image Lab 5.0 (Bio-Rad, Hercules, CA, USA). The concentrations of DNA marker bands were used as a reference.

### 2.6. Light Microscopy Assay of Herbal Drug (Potentillae argenteae herba)

**Figure 3:**
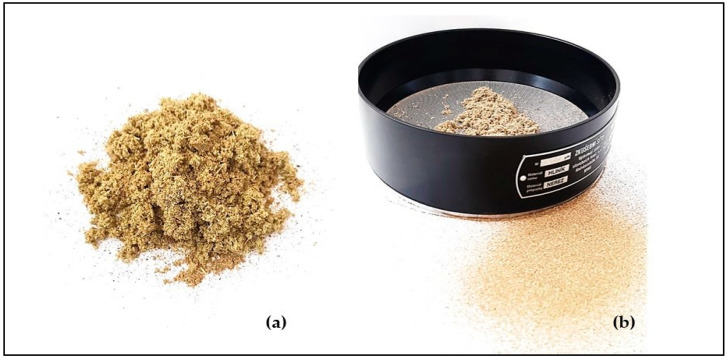
Powdered *Potentillae argenteae herba*—before (**a**), and after (**b**) sieving with a pharmacopoeial sieve.

The powdered aerial flowering parts of *P. argentea* were analyzed microscopically (in chloral hydrate solution) using a MagnumT trinocular light microscope CETI (Medline Scientific, Oxfordshire, UK) coupled with a photodocumentation system Si 5000 5Mpx (Medline Scientific, Oxfordshire, UK). The powder was sieved through a 0.4 mm pharmacopoeial sieve before microscopic examination (Figure 3).

### 2.7. Statistical Methods

Means and standard deviations for the chromatographic, phytochemical, and biological analyses were calculated using Microsoft Office Excel 2003. Results are expressed as the mean ± standard deviation (SD) based on three replicates. Statistical differences among the data were evaluated using analysis of variance (ANOVA) followed by Tukey’s HSD post hoc test, performed via the online tool Statistics Kingdom [24].

## 3. Results

### 3.1. Chemical Composition of the Dry Tincture (DT)

#### 3.1.1. GC-MS Identification

The obtained tincture was a dark brown dense mass with a smooth, glossy surface (Figure 2e), the yield of which was 6.8215 g dt/100 g dry plant material. The individual components of the DT, their quantitative values, and chromatographic peaks are presented in Table 1 and Figure S1. Eighteen volatiles were identified (constituting 98.95% of the total content), ranging from 1.44 to 39.38 mg/g dt. The predominant components (over 10 mg/g dt) in descending order were n-nonacosane, squalene, n-tricosane, ethyl oleate, and n-pentacosane. N-heptacosane, n-octadecane, ethyl linoleate, and *α*-tocopherol acetate were between 5 and 10 mg/g dt, and the remaining eight components represented below 5 mg/g dt.

From the results obtained it was evident that the chemical composition was dominated by aliphatic hydrocarbons, among which the most common was n-nonacosane, as well as their aliphatic oxygenated derivatives, mainly fatty acid esters—ethyl palmitate, ethyl oleate, ethyl linoleate, etc. Squalene was the only representative of the triterpene group (in significant amounts of 28 mg/g dt); neophytadiene, of the diterpenes; and o-acetylsalicylic acid, of the aromatic compounds in the composition.

#### 3.1.2. HPLC Phenolic Profile

The analyzed DT showed total polyphenols in the order of 440.78 mg GAE/g, which was an indicator of a high content of bioactive compounds and a reason to continue studies on their individual composition. Sixteen phenols, proven antioxidants, were used as standards. The presence of five phenols was demonstrated—two flavonoids and three phenolic acids (Table 2). As can be seen from the table, rosmarinic acid and flavonol glycoside rutin were the most abundant compounds with over 10 mg/g dt, followed by p-coumaric and salicylic acids, both with about 5 mg/g dt. The flavonol kaempferol was present in small quantities, and the aglycone quercetin was under the limit of quantification. Representative HPLC chromatograms of the identified phenolic compounds can be seen in Figure S2.

The phenolic profile of *P. argentea* DT indicates the presence of valuable phenolic compounds and proven antioxidants, such as glycoside rutin, rosmarinic, *p*-coumaric, and salicylic acids, which were detected in high amounts in the tincture.

### 3.2. Biological Activities Evaluation

#### 3.2.1. Antioxidant Capacity

The results obtained for the antioxidant activity of *P. argentea* DT are presented in Table 3. Two proven antioxidants (BHT and L-ascorbic acid) were used as positive controls. As can be seen from Table 3, significant results were detected by all four applied methods, with some differences between them in the degree of expression of this effect. The tincture’s ability to reduce cupric ions was the most pronounced, with the highest value being expressed (8617.54 μM TE/g dt). The capacity of DT to reduce ferrous ions was the next strongest (being almost three times lower than that of reducing copper ions), followed by the DPPH radical scavenger. The radical scavenging ability of the ABTS radical was less pronounced (2.4-fold) than that of DPPH.

The antioxidant capacity of *P. argentea* DT was confirmed in comparison to those obtained for the standards. Compared to BHT, the antioxidant capacity of the tincture was even more pronounced in three of the applied assays—CUPRAC, FRAP, and DPPH (Table 3). In the CUPRAC assay, the tincture also demonstrated a more powerful ability to reduce copper ions as compared to the other standard used, vitamin C.

The strong antioxidant effect is apparently due to the presence of high levels of polyphenols, as well as other proven antioxidants (such as alpha-tocopherol acetate, etc.) in the tincture.

#### 3.2.2. DNA Protective Capacity

**Figure 4:**
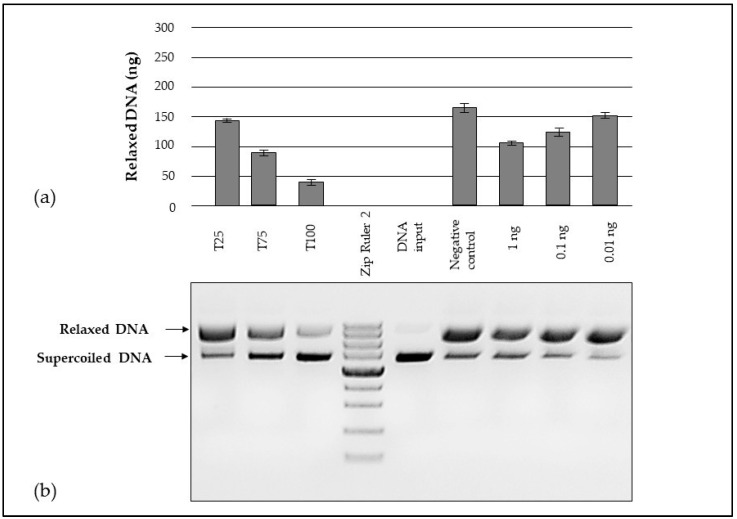
In vitro DNA protective capacity of *Potentillae argenteae herba* tincture: (**a**) relative concentration of relaxed plasmid DNA and (**b**) agarose gel electrophoresis. Lines T25, T75, and T100—different Trolox concentrations (25, 75, and 100 µg/mL); line Zip Ruler 2 Express DNA Ladder (Thermo Scientific, Waltham, MA USA); line plasmid DNA input; line of negative control; lines of dilutions of tested extract (1 ng; 0.1 ng; and 0.01 ng/mL). The line designations refer to parts (**a**,**b**).

The amount of relaxed DNA was used as a criterion for the DNA-protective properties of the tested tincture, obtained from the aerial flowering parts of the medicinal plant *P. argentea*. Since maintenance of supercoiled DNA topology requires an intact pentose-phosphate skeleton, any single-stranded nicking results in helix relaxation, which was visualized as a band mobility shift on agarose gel electrophoresis. Three concentrations were tested—an initial concentration of 1 ng/mL and subsequent ten-fold and hundred-fold dilutions. As illustrated in Figure 4, the band intensity negatively correlated with the amount of the extract supplemented. The protective effect was strongest at the highest dose of the tested extract (1 ng/mL). The result was similar for the Trolox positive control used.

The capacity of the tested tincture can be estimated as being similar to the capacity of 75 μg/mL Trolox.

### 3.3. Microscopic Diagnostic Features of the Potentillae argenteae herba

Microscopic identification has an important place in pharmacopeial monographs on herbal drugs. The powdered substance had a yellow-green color with a silvery tint (Figure 3).

**Figure 5:**
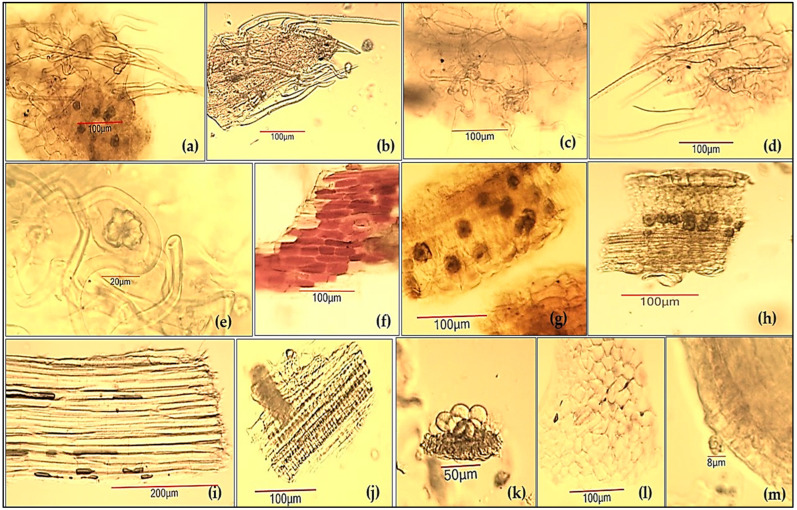
Micrographs of powdered *Potentillae argenteae herba*: (**a**) lower leaf epidermis with covering straight and curly unicellular trichomes with part of chlorenchyma tissue with oxalate druses; (**b**) fragment of sepal with covering trichomes; (**c**) fragment of petiole with covering trichomes; (**d**) fragment of flowering stalk with covering trichomes; (**e**) individual curly trichomes and oxalate crystals; (**f**) stem epidermis with reddish colored cells; (**g**) parenchyma tissue with cluster crystals; (**h**) leaf lamina in cross-section; (**i**) sclerenchyma fiber bundle; (**j**) fragment of conducting bundles with vessels; (**k**) pollen grains with fragment of the anther sheath; (**l**) petal fragment with papillary epidermis; (**m**) sessile gland with multicellular head on the leaf surface.

Examined under a microscope using chloral hydrate solution, the sieved powder showed the following diagnostic characters (Figure 5): unicellular covering trichomes—curly (silky) and straight (up to 500 μm), located on the lower leaf surface (a), on the sepals (b), the petiole (c), the apical part of the flowering stalk (d), and individual ones (e); fragments with epidermal cells (from the hairless part of the stalk) with a reddish content (f); cluster crystals of calcium oxalate (15–35 μm in diameter) included in the parenchyma tissue (a, g, h) and individual ones (b, e); fragments of leaf lamina in a cross-section, containing epidermal tissue, chlorenchyma tissue, oxalate crystals, and covering trichomes on the abaxial surface (h); groups of sclerenchymatous thick-walled fibers (i); fragments of spiral and reticulate vessels (j); fragments of anther with pollen grains (about 25 μm in diameter) (k); fragments of petals with papillary epidermis (l); and rare single glands (up to 10 μm in diameter) observed on the leaf surface (m).

Microscopic characterization of *Potentillae argenteae herba* has not been performed until now, which necessitated the more complete characterization of the substance; this showed that it clearly possesses the qualities of an herbal drug with a strong antioxidant effect.

## 4. Discussion

According to the information available in the literature, the yield obtained from *P. argentea* in this study was consistent with the data reported by Tomczyk et al. [5] and Paduh et al. [16]. They analyzed an aqueous extract from the above-ground parts, and reported a value of 6.80%, which corresponds to the ethanol tincture we obtained. Of the three types of extracts studied by Sut et al. [13], the yield of their aqueous extract was also of a similar value (6.46%), while that of methanol was about 2.8 times higher. The ethyl acetate extract of the same team had a two-fold lower yield (3.75%). The same three extractants (water, methanol, and ethyl acetate) were also preferred by another research team to prepare extracts of the species *P. speciosa* and *P. reptans* belonging to the genus Potentilla [8]. For Potentilla, Tomczyk et al. [25] reported yields of 1.98% to 5.03% for aqueous extracts from dried ground aerial portions.

Regarding GC/MS identification of chemical compounds in extracts, data are available only for a methanol extract obtained by ultrasound from the root of *P. argentea* [7]. Significant content of squalene was found; this is a component that we also detected in the ethanol extract of the aerial part of the same species. Squalene is a triterpene with important applications in medicine, cosmetics, and the food industry. It is a precursor of various bioactive compounds such as hormones, vitamins, cholesterol, sterol, and triterpenoids. This phytochemical can be used as a healing agent and has great therapeutic potential; for instance, it helps to reduce triglyceride and cholesterol levels and works as an antitumor agent in the treatment of various forms of cancer. Squalene facilitates cell oxidation, which leads to cell death due to hypoxia [26]. The predominant chemical component in the composition of the studied dry tincture, alkane nonacosane, was also present in the highest percentage among the identified phytochemical constituents of the medicinal species *Scutellaria orientalis*, with antimicrobial and anti-inflammatory activities indicated for this component [27]. Tricosane, which was among the major phytocompounds in the present chromatographic analysis, has been listed as an anticancer bioactive constituent [28]. It is associated with the significant decrease observed in the viability of tumor cells (cytotoxic activity against the HepG2 cell line) from the extract of *Boswellia serrata*, in which it was the main component. The authors point out that the extracts containing this natural antitumor agent are suitable for the production of phytopreparations for the treatment of oncological diseases The same bioactive compound, according to other studies, was associated with antioxidant and antibacterial activity exhibited against *Bacillus cereus*, *Staphylococcus aureus*, *Proteus vulgaris*, and *Klebsiella pneumoniae*, as it was predominant in the chemical composition of *Saba florida* root bark extract [29].

Fatty acid esters, such as ethyl palmitate and ethyl oleate, abundant in the studied tincture, are constituents of various types of emulsions used in the preparation of cosmetic and pharmaceutical products. The addition of ethyl oleate to microemulsions increases the solubility of drugs and leads to better bioavailability by improving lymphatic transport; for example, piroxicam dissolves 30 times better in ethyl oleate than in water [30].

Alpha-tocopherol acetate (also called vitamin E acetate), which was identified in *P. argentea* DT in good amounts, is another essential ingredient in cosmetic products and dermatological preparations. The compound acts as a proactive drug that slowly releases active vitamin E. Topically applied, *α*-tocopherol acetate provides protection to the skin against harmful sun effects, proven by in vivo studies [31]. This form of vitamin E also inhibits the adhesion of various bacterial pathogens capable of forming a biofilm on various surfaces of hospital equipment [32]. Therefore, esterified tocopherol could be a promising molecule to coat the surfaces of implants and thus limit possible infections. Neuroprotective activity, immunomodulatory function, protection against allergic conditions, and other beneficial effects have been proven for *α*-tocopherol. This vitamin is related to the proper development of the brain, muscles, skin, etc. [33]. Vitamin E, as an antioxidant, also protects cell membranes from oxidative damage [34].

Total polyphenol content (TPC) in extracts from above-ground parts of *P. argentea* has also been the subject of research by other authors, who have analyzed mainly aqueous, methanol, and ethyl acetate extracts. According to Augustynowicz et al. [6], the total polyphenols in a methanol extract amounted to 339.72 mg GAE/g extract (close to, but lower than, the amount obtained in the present study), in contrast to those reported by Sut et al. [13], which were several times lower (17.44–113.29 mg GAE/g extract). TPCs many times lower (over 7 times), determined by Folin–Ciocalteu’s method, were also reported by Tomczyk et al. [5] and Paduch et al. [16] for an aqueous extract. Lower than the data in the current study are those reported by Uysal et al. [9] for methanol extracts, but obtained from the roots of the plant species (133.45 mg GAE/g extract). The higher amounts of total polyphenols identified by our team could be attributed to the use of ethanol as a solvent, as well as to the geographical and climatic conditions, which appear to be favorable for the accumulation of more bioactive compounds.

There is scarce research reported in the literature on the individual phenolic composition of *P. argentea.* In an extract from the above-ground parts of the species, but using methanol as a solvent, Sut et al. [13] identified (HPLC-DAD-MS technique) caffeic acid derivatives with a content of 3.28 mg/g. In our ethanol tincture, this phenolic acid was not detected. The authors also reported a content of ellagic acids—12.55 mg/g, ellagitannin derivatives (0.46–39.67 mg/g), and five flavonoid glycosides (0.35–24.62 mg/g), among which quercetin glucuronide (24.62 mg/g) had the highest content. The difference in chemical profile can be attributed to both the solvent used to obtain the extracts and the different methodology, which affect the ability to extract the bioactive compounds from the plant parts. The flavonoid rutin, which we detected at over 10 mg/g dt in the dry tincture, was not identified in the ethyl acetate dry extract of *P. argentea* aerial parts analyzed by Tomczyk et al. [15]. Of the four phenolic standards in the study (rutin, isoquercitrin, hyperoside, and quercetin 3-glucuronide), they found only quercetin 3-glucuronide (31.1 mg/g, *w*/*w*) for the species. Using the HPLC-DAD-MS method, Hribova et al. [14] determined the presence of quercetin derivatives, epigallocatechin gallate, galloyl-glucose, and tiliroside, and the absence of routine, in an aqueous extract from above-ground flowering portions of *P. argentea*.

Recent studies by Augustynowicz et al. [6] (MS and UV-Vis techniques) detected some phenolic acids and flavonoids in an acetone extract of *P. argentea*. They identified gallic acid and its derivatives, as well as catechin, which we did not find. They also found quercetin derivatives, while in the current experiment, the flavonoid quercetin was below the limit of quantification. In addition, ellagic acid and its derivatives, biliroside (cis and trans forms), isorhamnetin, kaempferol derivatives, and other phenolic compounds have also been determined qualitatively but not quantitatively. Uysal et al. [8] reported quantitative values for phenolics for two other species of the genus Potentilla (*P. reptans* and *P. speciosa*), where rutin is the predominant component in three extract types (ethyl acetate, methanol, and aqueous). In *P. speciosa*, this flavonoid glycoside had a value close to that determined by us (9.21 mg/g extract for methanol and 11.01 mg/g extract for aqueous extracts). The authors indicate significantly lower quantitative values for *p*-coumaric acid (0.01–0.29 mg/g extract), which in the ethanol tincture of *P. argentea* was one of the predominant phenolic acids in our study. This phenolic acid has been indicated as part of the phenolic composition of another medicinal species of the same genus—*P. recta*—but in a much smaller amount (over 230 times for methanol extract) [35]. The latter authors found the presence of vanillic, chlorogenic, and syringic acids (0.341 mg/g, 0.008 mg/g, and 0.004 mg/g, respectively), which were absent in *P. argentea.*

Rosmarinic acid, which was in the highest proportion in the present tincture, has shown significant therapeutic potential. An overview describes its potential antidiabetic, antimicrobial, cardioprotective, antiallergic, antidepressant, nephroprotective, hepatoprotective, anti-inflammatory, antiaging, and anticancer functions [36]. Therefore, this phenolic component can be considered as part of the treatment for a number of diseases. Among the hydroxycinnamic acid derivatives studied, rosmarinic acid is the most potent antioxidant. This phenolic acid is involved in the regulation of the enzyme glutamate cysteine ligase. In vivo studies have shown its influence on oxidized/reduced glutathione in the serum of experimental mice (its direct influence on redox homeostasis has been demonstrated). Induction of apoptosis in cancer cells, inhibition of cell proliferation, reduction in metastasis, suppression of cell adhesion, inhibition of pro-inflammatory cytokines and microRNAs, etc., are just some of the its different mechanisms of action. The flavonoid rutin was also found in the same amount as rosmarinic acid. The growing interest in this flavonol glycoside and its possible plant sources in recent years is based on its manifold benefits for human well-being. The development of methods and techniques for its isolation, as well as those aimed at increasing its bioavailability in the body, has been the focus of various scientific studies. This promising flavonoid is a key ingredient in nutritional supplements for the prevention and treatment of various chronic diseases in humans. Information summarized by Gullon et al. [37] reveals the pharmacological properties of rutin: antioxidant, antidiabetic, anti-inflammatory, antimicrobial, anticancer, antiallergic, and other biological activities (treatment of Parkinson’s disease and Alzheimer’s disease; antihypertensive, antithrombogenic, and antidepressant activities; treatment of chronic venous insufficiency, etc.). *P*-coumaric acid, which is present in the tincture, also deserves attention. Pharmacological studies focus on its positive effect in diseases related to oxidative stress—neurodegenerative and cardiovascular diseases, inflammatory processes, etc. [38]. The various mechanisms with which it suppresses oxidative stress and enhances the healing powers of the body have been studied. In vivo and in vitro tests have proved its antioxidant properties (neutralizes free radicals and reduces the resulting DNA oxidative damage). Molecular mechanisms have revealed its immunoregulatory, anti-inflammatory, neuroprotective, antitumor, antibacterial, anti-aging, and other biological activities.

The antioxidant potential of the medicinal species *P. argentea* was investigated by Sut et al. [13] using the same four methods for three different types of above-ground plant extracts. The strongest effect was obtained by the DPPH method (319.60 mg TE/g extract) for a methanol extract, and the lowest by the ABTS method (1.27 mg TE/g extract) for an ethyl acetate extract. For their methanol extract, the following descending order was observed, depending on the strength of the activity: DPPH > CUPRAC > FRAP > ABTS, while for aqueous and ethyl acetate extracts, the arrangement was similar to the current experiment (CUPRAC > FRAP > DPPH > ABTS). The above authors also reported the lowest activity obtained by the ABTS method for all three extracts. Methanol and aqueous extracts have significantly higher quantitative indicators compared to ethyl acetate extract (a tendency observed with all four methods applied). Paduh et al. [16] reported the strongest antioxidant activity (DPPH method) of aqueous extracts from above-ground portions of species of the genus Potentilla. They identified *P. argentea* as the most potent in radical reduction (60% of the radicals were reduced at an extract concentration of 75 μg/mL compared to the control). According to the same authors, the high levels of measured phenolic compounds (total phenolic content, total phenolic acids content, total flavonoids content, total tannins content, and total proanthocyanidins content) were responsible for the high activity of selected Potentilla species. Besides the phenolic compounds in the composition, the type of solvent used is significant for the antioxidant power of the extract and for determining the optimal conditions for their solubility in order to increase their activity. Condensed tannins were responsible for the inhibition of the DPPH radical (over 95%) from water–methanol extracts derived from *P. argentea* (herba and rhizomes) [17]. The observed AOA was reduced by half after the removal of the ethyl acetate-soluble condensed tannins. Research by Uysal et al. [8] added more information about the antioxidant potential of extracts (ethyl acetate, methanol, and aqueous) of other representatives of the genus Potentilla (*P. speciosa* and *P. reptans*) as well. For an aqueous extract of *P. speciosa*, the authors’ team found a similar one to the one mentioned above by Sut et al. [13] in descending order for *P. argentea* (CUPRAC > FRAP > DPPH > ABTS) according to the strength of the activity measured (4.17–269.18 mg TE/g extract). For a methanol extract of *P. speciosa*, the strongest is the radical scavenging effect reported by the DPPH method (334.66 mg TE/g). The second species analyzed by the authors, *P. reptans*, showed activity ranging from 2.06 to 292.10 mg TE/g extract. In our study, the antioxidant value of *P. argentea* DT, obtained by CUPRAC assay, was significantly higher than those obtained by the other methods. The activity of the investigated extract (8617.54 a ± 225.71) was also higher than that of vitamin C (7492.40 b ± 88.87) and BHT (3499.42 c ± 198.29). This could be explained by the complex nature of *P. argentea* DT extract, which was rich in various groups of bioactive compounds, including phenolic acids (rosmarinic acid), flavonoids (rutin), and volatiles (squalene). CUPRAC assay is the most sensitive antioxidant method, based on electron transfer (with lower reduction potential than FRAP), and is the only assay which is conducted on physiological pH; additionally, the redox reaction goes to the endpoint for most flavonoids and it can assay –SH bearing antioxidants without interfering with citric acid and sugars.

The high antioxidant potential characteristic of phenolic compounds quite logically explains the subsequent positive result for the DNA protective effect of the extract of the medicinal species. A similar DNA effect has been reported for plant extracts from other species—*Coronilla varia*, *Semecarpus kurzii*, *Moringa oleifera*, etc. [39,40,41]. They also tested the protection of DNA from damage induced by Fenton’s reagent. Such molecular studies point to the existing direct relationship between the high concentration of phenolic substances and the ability of plant extracts to counteract DNA damage. In this line of thought, specifically for *p*-coumaric acid, there is evidence of its direct role in reversing DNA damage caused by oxidative stress [38]. This bioactive ingredient is the second most abundant phenolic acid in the composition, suggesting that its presence probably contributes to the observed biological effect. The effectiveness of *P. argentea* extracts in removing DNA damage caused by oxidative stress has not been reported before.

## 5. Conclusions

The present study reports data on the chemical composition and biological activities of *Potentillae argenteae herba* ethanol dry tincture for the first time. Among the gas chromatographically determined 18 volatile compounds, n-nonacosane, squalene, n-tricosane, ethyl oleate, and n-pentacosane were in the highest amounts (in the range of 40 to 10 mg/g dt). The individual phenolic composition showed the presence of rutin, kaempferol, rosmarinic acid, *p*-coumaric acid, and salicylic acid, of which rosmarinic and salicylic acids have not been reported before for the species. The antioxidant potential of the tincture was strongly expressed and confirmed by comparison to proven antioxidants such as butylhydroxytoluene and vitamin C. The DNA protective activity was newly found for silver cinquefoil. Microscopic characterization of the powdered aerial parts of *P. argentea* has not been undertaken before and is an important part of the pharmacognostic analysis of herbal drugs. The dry tincture has the potential to be an effective herbal product for the prevention and treatment of diseases caused by free radicals (neurodegenerative, cardiovascular, inflammatory, etc.). It could also be a source of valuable bioactive components such as squalene, rutin, and rosmarinic acid. Future studies about potential toxicity are needed to determine the application form and safety of the tincture.

## Figures and Tables

**Table 1:** Chemical composition of *Potentilla argentea* DT (mean ± SD).

Peak	RT	RI_calc_	RI_lit_	Compound	Content (mg/g dt)
1	12.34	1802	1800	n-Octadecane	7.64 ^h^ ± 0.04
2	12.72	1837	1841	Neophytadiene	2.36 ^n^ ± 0.15
3	13.02	1870	1872	o-Acetylsalicylic acid 1TMS	3.85 ^l^ ± 0.05
4	14.21	1902	1900	n-Nonadecane	1.44 ^o^ ± 0.15
5	14.78	1958	1960	Ethyl palmitate	14.50 ^e^ ± 0.10
6	17.49	2162	2163	Ethyl linoleate	6.56 ^i^ ± 0.17
7	17.58	2171	2169	Ethyl oleate	15.24 ^d^ ± 0.06
8	18.04	2195	2192	Ethyl stearate	4.71 ^j^ ± 0.06
9	19.78	2303	2300	n-Tricosane	18.36 ^c^ ± 0.14
10	21.24	2390	2392	Ethyl arachidate	1.44 ^o^ ± 0.04
11	21.35	2398	2400	n-Tetracosane	3.21 ^m^ ± 0.01
12	22.90	2501	2500	n-Pentacosane	10.60 ^f^ ± 0.10
13	24.30	2593	2590	Ethyl docosanoate	2.50 ^n^ ± 0.14
14	25.84	2702	2700	n-Heptacosane	8.94 ^g^ ± 0.07
15	27.42	2817	2814	Squalene	28.88 ^b^ ± 0.18
16	28.63	2901	2900	n-Nonacosane	39.38 ^a^ ± 0.18
17	31.23	3100	3100	n-Hentriacontane	4.31 ^k^ ± 0.03
18	31.52	3131	3140	*α*-Tocopherol acetate	6.62 ^i^ ± 0.04
**Total identified compounds, %**				**98.95**	

RT refers to retention time; RI calc represents the relative retention index as calculated by the authors, while RI lit denotes the relative retention index from literature sources. The data are presented as means ± standard deviation (SD) and are based on triplicate measurements. Significant differences among the measured features, indicated by distinct small superscript letters, were determined using Tukey’s test at a significance level of *p* < 0.01.

**Table 2:** Total and individual phenolic content in *Potentilla argentea* DT (mean ± SD).

№	Compounds	Content, mg/g dt
**Flavonoids**		
1	Rutin	10.52 ^a^ ± 0.16
2	Hesperidin	NF *
3	Quercetin	ULOQ **
4	Kaempferol	0.07 ^c^ ± 0.03
5	(+)-Catechin	NF
6	(−)-Epicatechin	NF
**Phenolic acids**		
7	Gallic acid	NF
8	Protocatechuic acid	NF
9	Vanillic acid	NF
10	Syringic acid	NF
11	*p*-Coumaric acid	5.54 ^b^ ± 1.44
12	Salicylic acid	4.99 ^b^ ± 0.19
13	Chlorogenic acid	NF
14	Caffeic acid	NF
15	Ferulic acid	NF
16	Rosmarinic acid	10.83 ^a^ ± 0.01
**Total phenolic content (Folin–Ciocalteu)**		440.78 ± 59.67 mg GAE/g dt ***

* NF—not found; ** ULOQ—under the limit of quantification; *** mg GAE/g dt—milligrams of gallic acid equivalents per gram of dry tincture. The data, derived from three replicates, are shown as means ± standard deviation (SD). Means denoted by different small superscript letters are significantly different as per Tukey’s test (*p* < 0.01).

**Table 3:** In vitro antioxidant capacity of *Potentilla argentea* DT (mean ± SD).

Sample	ABTS-Assay μM TE/g dt	DPPH-Assay μM TE/g dt	FRAP-Assay μM TE/g dt	CUPRAC-Assay μM TE/g dt
*P. argentea* DT	938.35 ^c^ ± 60.08	2258.53 ^b^ ± 109.47	3025.71 ^b^ ± 126.20	8617.54 ^a^ ± 225.71
BHT	1781.25 ^b^ ± 157.37	1900.29 ^c^ ± 82.92	1794.57 ^c^ ± 41.11	3499.42 ^c^ ± 198.29
L-Ascorbic acid	2762.72 ^a^ ± 114.93	7471.97 ^a^ ± 167.70	8197.22 ^a^ ± 98.78	7492.40 ^b^ ± 88.87

μM TE/g dt—micromoles of Trolox equivalents per gram of dry tincture; ABTS radical scavenging assay; DPPH radical scavenging assay; FRAP—ferric reducing antioxidant power assay; CUPRAC—cupric reducing antioxidant capacity assay; BHT—butylated hydroxytoluene. Results are reported from triplicate measurements. Distinct small superscript letters within the columns indicate significant differences according to Tukey’s test (*p* < 0.01).

## Data Availability

The original contributions presented in this study are included in the article. Further inquiries can be directed to the corresponding author.

## Supplementary Materials

The following supporting information can be downloaded at: https://www.mdpi.com/article/10.3390/antiox14040487/s1, Figure S1: GC/MS chromatogram of the volatile compounds from in *Potentillae argenteae herba* ethanol dry tincture: 1—n-Octadecane; 2—Neophytadiene; 3—o-Acetylsalicylic acid 1TMS; 4—n-Nonadecane; 5—Ethyl palmitate; 6—Ethyl linoleate; 7—Ethyl oleate; 8—Ethyl stearate; 9—n-Tricosane; 10—Ethyl arachidate; 11—n-Tetracosane; 12—n-Pentacosane; 13—Ethyl docosanoate; 14—n-Heptacosane; 15—Squalene; 16—n-Nonacosane; 17—n-Hentriacontane; 18—*α*-Tocopherol acetate; Figure S2: Typical HPLC chromatograms (280 nm and 360 nm) of the phenolic acids and flavonoids in *Potentillae argenteae herba* dry tincture (A) and mixture of standards (B). 1—Gallic acid, 2—Protocatechuic acid, 3—(+)-Catechin, 4—Chlorogenic acid, 5—Vanillic acid, 6—Caffeic acid, 7—Syringic acid, 8—(-)-Epicatechin, 9—p-Coumaric acid, 10—Ferulic acid, 11—Salicylic acid, 12—Rutin, 13—Hesperidin, 14—Rosmarinic acid, 15—Quercetin, 16—Kaempherol.
