# Supplementary material for: Potentillae argenteae herba—Antioxidant and DNA-Protective Activities, and Microscopic Characters
Source: Antioxidants (Basel). 2025 Apr 18;14(4):487. doi: 10.3390/antiox14040487 (PMC12023972; doi:10.3390/antiox14040487)
Supplement: Supplementary file 1 [file antioxidants-14-00487-s001.zip › Figure S1.pdf]

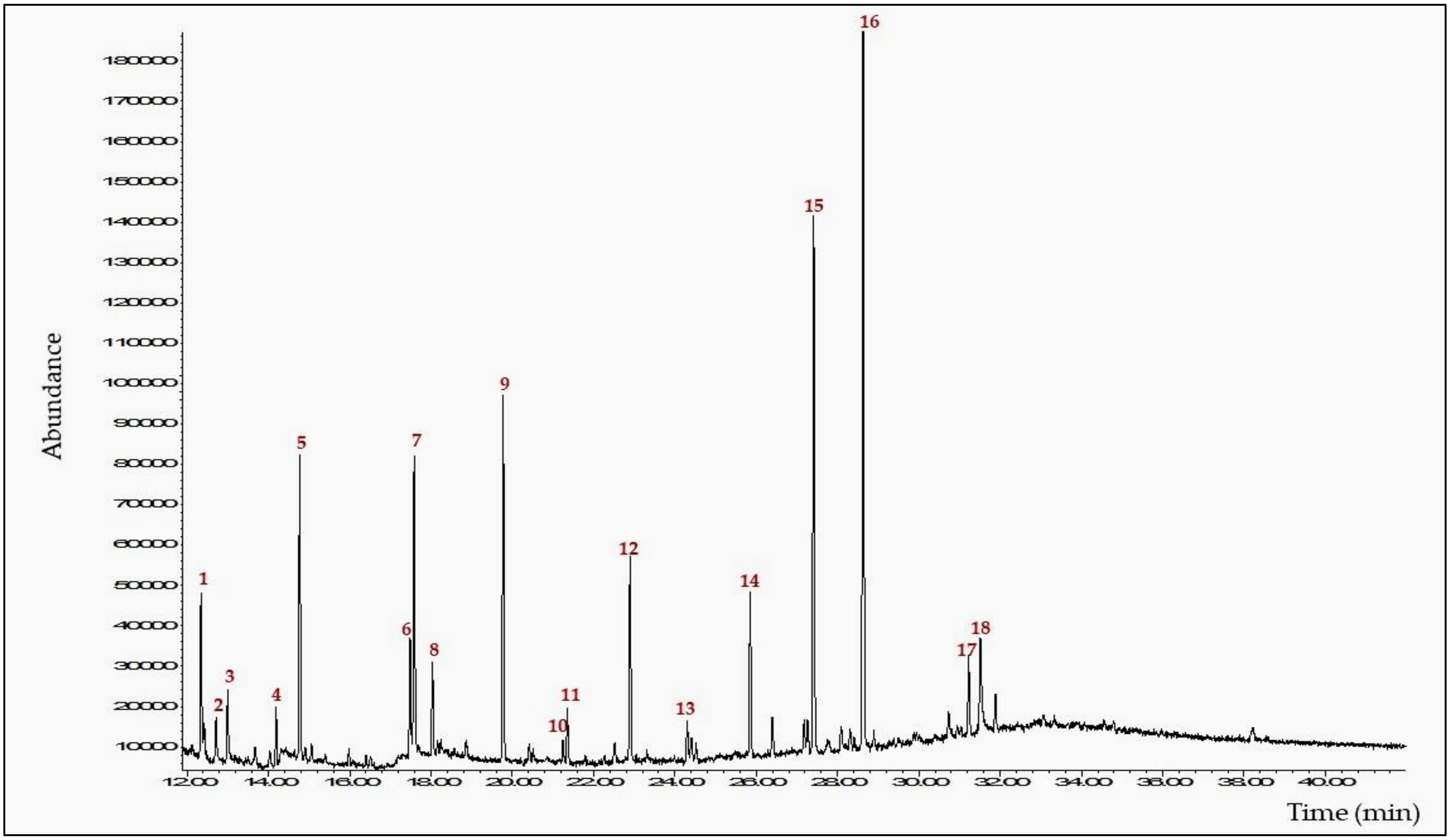

Figure S1. GC/MS chromatogram of the volatile compounds from in *Potentillae argenteae herba* ethanol dry tincture: 1 - n-Octadecane; 2 – Neophytadiene; 3 - o-Acetylsalicylic acid; 4 - n-Nonadecane; 5 - Ethyl palmitate; 6 - Ethyl linoleate; 7 - Ethyl oleate; 8 - Ethyl stearate; 9 - n-Tricosane; 10 - Ethyl arachidate; 11 - n-Tetracosane; 12 - n-Pentacosane; 13 - Ethyl docosanoate; 14 - n-Heptacosane; 15 – Squalene; 16 - n-Nonacosane; 17 - n-Hentriacontane; 18 - α-Tocopherol acetate.
