# Supplementary material for: Potentillae argenteae herba—Antioxidant and DNA-Protective Activities, and Microscopic Characters
Source: Antioxidants (Basel). 2025 Apr 18;14(4):487. doi: 10.3390/antiox14040487 (PMC12023972; doi:10.3390/antiox14040487)
Supplement: Supplementary file 1 [file antioxidants-14-00487-s001.zip › Figure S2.pdf]

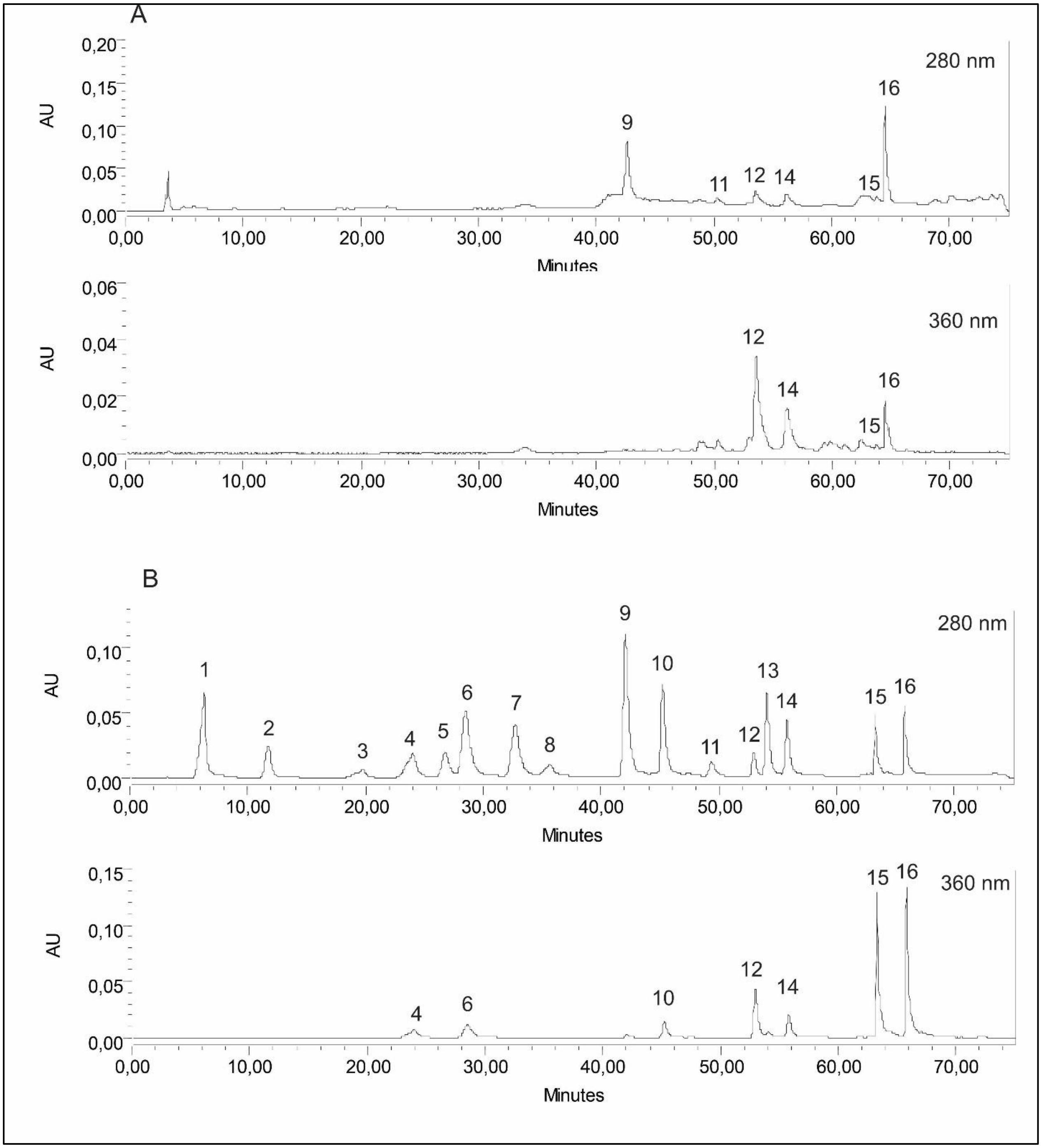

Figure S2. Typical HPLC chromatograms (280 nm and 360 nm) of the phenolic and flavonoids in *Potentillae argenteae herba* dry tincture (A) and mixture of standards (B). 1 – Gallic acid, 2 - Protocatehuic acid, 3 - (+)-Catechin, 4 - Chlorogenic acid, 5 - Vanillic acid, 6 - Caffeic acid, 7 - Syringic acid, 8 - (-)-Epicatechin, 9 - p-Coumaric acid, 10 – Ferulic acid, 11 - Salicylic acid, 12 - Rutin, 13 - Hesperidin, 14 - Rosmarinic acid, 15 - Quercetin, 16 – Kaempherol
